# Invagination intestinale aiguë consécutive à un lipome grèlique: à propos d'un cas et revue de la littérature

**Published:** 2012-08-07

**Authors:** Kamal Bentama, Iliass Chemlal, Mohamed Benabbou, Mohamed El Abssi, Meouhamed El Ouananni, Mouhamed Cherrab, Alami Faricha, Abdelkader Errougani, Mohamed Amraoui, Rachid Chekoff

**Affiliations:** 1 Service des Urgences Chirurgicales Viscérales (Pr. CHEKOFF RACHID), Hôpital Ibn Sina, Rabat, Maroc

**Keywords:** Invagination, grele, lipome, intussusception, small intestine, lipoma

## Abstract

L'invagination intestinale aiguë est une pathologie du nourrisson et du petit enfant. Sa survenue chez l'adulte est très inhabituelle. Elle est d’étiologie diverse. Dans l'immense majorité des cas, elle est secondaire à une tumeur qui peut être bénigne ou maligne. L'invagination intestinale sur lipome est exceptionnelle. Nous rapportons un cas d'invagination intestinale grêlo-grêlique sur lipome.

## Introduction

L'invagination iléale aiguë (IIA) de l'adulte, à la différence de l'enfant, est une manifestation rare survenant le plus souvent au cours d'une tumeur du grêle d'origine maligne. Elle représente 1 à 5% des étiologies d'occlusion intestinale chez l'adulte [1]. Son mode évolutif est habituellement chronique ou subaigu [2–4]. Elle est rarement découverte devant un tableau aigu d'occlusion intestinale ou de péritonite [5]. Chez l'adulte une cause organique est trouvée dans 70 à 90% des cas, alors que, chez l'enfant l'invagination intestinale est le plus souvent idiopathique [2, 6]. En conséquence chez l'adulte, le traitement est chirurgical fondé sur la résection intestinale avec cependant un débat encore ouvert concernant la nécessité ou non d'une réduction préalable du boudin d'invagination [1, 6]. [Agrave] partir de ce nouveau cas et après analyse de la littérature, nous discutons les caractéristiques cliniques, diagnostiques et les possibilités thérapeutiques de cette pathologie rare.

## Observation

EL K. A., âgé de 37 ans, sans antécédent particulier, était hospitalisé le 11 juin 2011 pour des douleurs abdominales diffuses avec notion d'arrêt des matières et des gaz et de vomissements fécaloïdes. Le début de sa symptomatologie clinique remontait à deux mois par la survenue de douleurs abdominales paroxystiques diffuses à type de crampes avec vomissements. Son transit s’était modifié avec une tendance à la constipation, parfois associée à des selles liquides. Ce syndrome abdominal a été résolutif puis entrecoupé d’épisodes douloureux paroxystiques jusqu'au jour de son hospitalisation motivée par l'accentuation des douleurs et l'arrêt des matières et des gaz. A l'admission, L'examen clinique objective un abdomen distendu avec tympanisme à la percussion, légèrement sensible, sans masse palpable, les orifices herniaires étaient libres. Le patient était apyrétique. Il n'existait pas d'altération récente de l’état général. Le toucher rectal était normal. Le reste de l'examen clinique était normal. Les examens biologiques usuels étaient sans particularité.

**Figure 1:**
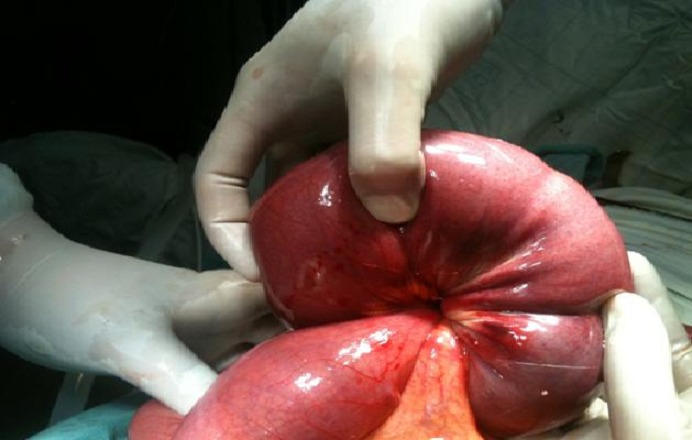
Image opératoire de l'invagination intestinale aiguë de notre patient, à noter l'absence de lésions suspecte en surface

La radiographie de l'abdomen sans préparation montrait quelques niveaux hydroaériques sur l'intestin grêle. Le scanner abdominopelvien montrait la présence d'un syndrome occlusif mécanique de l'ensemble du grêle sur une probable invagination iléo-iléale, avec une image du boudin d'invagination, sans montrer de cause. L'indication opératoire était formelle. L'intervention chirurgicale, menée par une laparotomie médiane a chevale sur l'ombilic, a permis de confirmer la présence d'une IIA située à environ 70–80 cm de l'angle duodéno-jéjunale, avec une nécrose en partie du boudin d'invagination (Figure 1). L'intestin en aval était plat alors qu'en amont, il était dilaté avec une hypertrophie de la paroi intestinale. Il n'existait pas d'adénopathie mésentérique. Une résection de 50–60 cm d'intestin grêle était pratiquée enlevant les zones ischémiques, avec anastomose immédiate. L’étude anatomopathologique est en faveur d'un lipome sous muqueux. Les suites opératoires ont été simples et le patient a quitté l'hôpital au 8e jour de son hospitalisation.

## Discussion

L'invagination intestinale est définie par le télescopage et la pénétration d'un segment intestinal (anse invaginée) dans le segment d'aval (anse réceptrice). Elle détermine un tableau occlusif, potentiellement grave en raison du risque d'ischémie intestinale. Elle survient essentiellement chez le nourrisson (80% entre 6 mois et 2 ans) [1]. L'atteinte de l'adulte, comme dans notre cas, est rare [1]. En Selon les publications, seulement 1 et 5% des IIA s'observent chez l'adulte, contre plus de 95% chez l'enfant. La différence ne s'arrête pas là, puisque chez l'enfant, l'IIA survient le plus souvent au cours d'une pathologie bénigne (adénolymphite mésentérique) et ne nécessite pas, la plupart du temps d'intervention chirurgicale. En revanche, chez l'adulte, l'IIA est le plus souvent en rapport avec une pathologie tumorale maligne (jusque dans 64% des cas) ou bénigne. La pathologie tumorale maligne est la première étiologie en cause. L’étiologie lipomateuse, cas de notre observation, est exceptionnelle. En effet, 28 cas seulement sont rapportés dans la littérature. Le lipome est une lésion rare du tube digestif. Ils atteignent électivement l'iléon au voisinage de la valve iléo-cæcale et le jéjunum proximal. La tumeur, de siège initial sous muqueux, se développe en direction de la lumière en repoussant la muqueuse. Ces tumeurs sont en général asymptomatiques. L'apparition de manifestations cliniques est corrélée à leur taille (à partir de 4cm) responsable alors de douleurs aiguës, de saignements occultes par ulcération de la muqueuse et d'invagination intestinale [4].

L'IIA peut également survenir au niveau du côlon (iléo colique), localisation digestive où les pathologies tumorales malignes sont encore plus fréquentes. L'invagination intestinale de l'adulte peut être difficile à reconnaître, d'autant plus qu'elle peut évoluer selon plusieurs tableau clinique: tableau occlusif aigu, tableau subocclusif de survenue progressive s’étendant de quelques jours à quelques semaines, syndromes abdominaux non spécifiques (modification du transit, douleurs abdominales diffuses, saignements digestifs,...) évoluant parfois pendant plusieurs mois avec ou sans altération de l’état général. Dans le cas de la pathologie lipomateuse, cas de notre observation, L'apparition de manifestations cliniques est corrélée à leur taille (à partir de 4 cm) responsable alors de douleurs aiguës, de saignements occultes par ulcération de la muqueuse et d'invagination intestinale [4]. Dans notre cas la symptomatologie clinique était faite par des douleurs paroxystique, des épisodes de subocclusion a répétition et enfin par une IIA. Quelle que soit la présentation clinique initiale, le diagnostic se fait majoritairement par l'imagerie (échographie, scanner), plus rarement par la chirurgie exploratrice. Sur le plan radiologique, les radiographies de l'abdomen sans préparation peuvent contribuer à poser le diagnostic d'occlusion de l'intestin grêle, la visualisation directe de la tête du boudin sous forme d'une masse de tonalité hydrique moulée par de l'air du segment intestinal d'aval est très rare [1]; mais dans la plupart des cas, cet examen fournit peu de renseignements.

**Figure 2:**
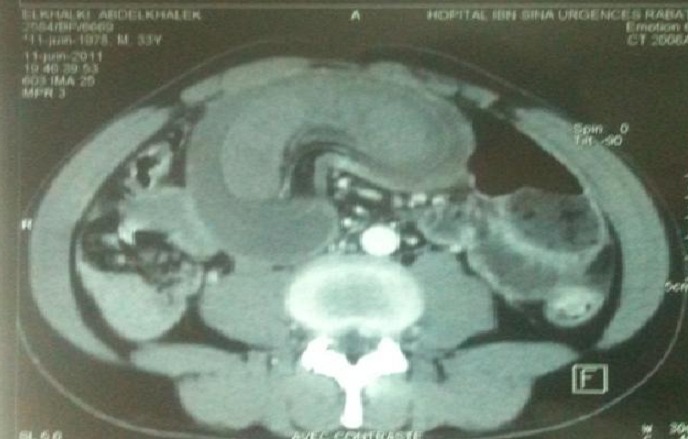
Scanner abdominal C(+), montrant l'image typique du boudin d'invagination

**Figure 3:**
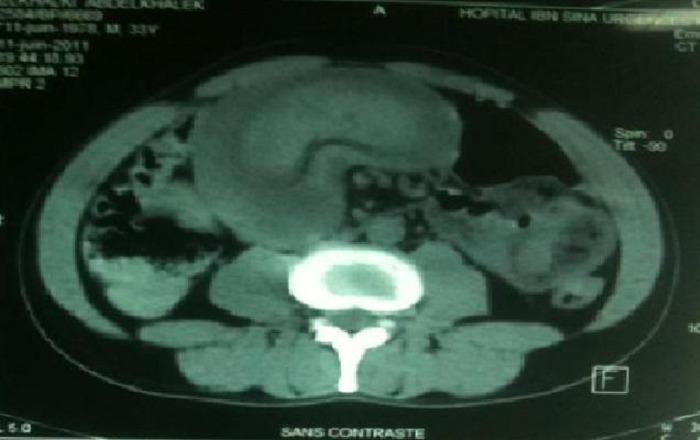
Image scannographique C(-) montrant l'image en cocard

L’échographie abdominale est un examen fiable et paraît prometteur pour le diagnostic d'invagination intestinale [4, 5], elle donne typiquement en coupe longitudinale une image en cible avec deux anneaux hypoéchogènes périphériques et un anneau central échogène, et en coupe transversale [4, 5] une image en «sandwich» avec trois cylindres superposés, qui correspond au boudin d'invagination. La tomodensitométrie réalisée en urgence, permet d'augmenter la sensibilité du diagnostic. Elle est plus performante que l’échographie. Il permet de diagnostiquer le syndrome obstructif, son mécanisme, en l'occurrence, l'invagination, sa localisation précise les signes de souffrance intestinale [4, 6] et de montrer sa cause (masse intraluminale ou extraluminale) [4]. En cas de lipome, il met en évidence une lésion intraluminale de densité graisseuse au centre entourée d'une paroi digestive. Elle peut détecter une cause organique dans 71% des cas [6, 7]. Les deux images classiques sont l'image « en sandwich » en coupe longitudinale dessinant la tête de l'IIA et l'image « en cocarde » en coupe transversale montrant le boudin de l'IIA (Figure 2, Figure 3). Dans notre cas, le scanner a été d'un grand apport; il a permis la mise en évidence du boudin d'invagination sous l image typique en cocard. Lorsque le diagnostic est suspecté, une intervention chirurgicale en urgence ou semiurgence doit être pratiquée, selon l’état du patient afin d'arriver au diagnostic par l'analyse anatomopathologique de la pièce opératoire [5, 7]. Dans cette observation, le scanner abdominal ainsi que la laparotomie ont permis de confirmer le diagnostic.

Le traitement est toujours chirurgical chez l'adulte et ne laisse aucune place à la réduction par hyperpression sous contrôle radiologique, étant donné la fréquence des causes organiques sous-jacentes. Une résection plus ou moins étendue peut être nécessaire [8]. Le recours à une simple désinvagination est licite dans les formes idiopathiques. L'exérèse intestinale selon les règles carcinologiques s'impose lors de la découverte d'une tumeur à l’évidence maligne. Le geste chirurgical est choisi en fonction de la présence ou non d'une tumeur et d'une nécrose intestinale. Une désinvagination par expression manuelle du boudin a est le geste exclusif face à une invagination iléo-iléale en l'absence de nécrose et de tumeur. Une résection intestinale doit être pratiquée dans les autres cas. Elle doit être faite sans désinvagination préalable en raison, soit de la nécrose du boudin, soit du caractère serré de l'invagination (cas de notre observation; en effet, dans ces cas la tentative de désinvagination expose à un risque de perforation intestinale. Cependant la désinvagination préalable, lorsqu'elle est possible, permet de mieux apprécier les limites de la résection et parfois de réduire son étendue notamment en cas de tumeur bénigne [3]. C'est dire la difficulté du choix thérapeutique adéquat qui doit tenir compte des découvertes opératoires et du résultat fonctionnel prévisible après la résection. Ainsi, quand la longueur du segment à réséquer peut exposer à un syndrome du grêle court, une désinvagination préalable devrait être tentée [9].

L’étude anatomopathologique est nécessaire pour la confirmation diagnostique et doit être complétée dans certains cas par une étude immunohistochimique (le cas des lymphomes).

## Conclusion

L'invagination grêlo-grélique secondaire à un lipome intestinal est rare et liée directement a la taille du lipome. L'imagerie dominée essentiellement par l’échographie et le scanner qui permet un diagnostic positif et surtout étiologique de l'affection en montrant des images évocatrices. La tomodensitométrie permet de confirmer la nature graisseuse du lipome.
